# Fog-Harvesting Properties of *Dryopteris marginata*: Role of Interscalar Microchannels in Water-Channeling

**DOI:** 10.3390/biomimetics3020007

**Published:** 2018-04-12

**Authors:** Vipul Sharma, Ramachandran Balaji, Venkata Krishnan

**Affiliations:** School of Basic Sciences and Advanced Materials Research Center, Indian Institute of Technology Mandi, Kamand, Mandi 175005, Himachal Pradesh, India; vipulsharma.cba@gmail.com (V.S.); balajiyashik@gmail.com (R.B.)

**Keywords:** fog-harvesting, water-channeling, biomimetics, soft lithography

## Abstract

Several flora and fauna species found in arid areas have adapted themselves to collect water by developing unique structures and to intake the collected moisture. Apart from the capture of the moisture and fog on the surface, water transport and collection both play an important part in fog-harvesting systems as it prevents the loss of captured water through evaporation and makes the surface available for the capture of water again. Here, we report the remarkable fog collection and water-channeling properties of *Dryopteris marginata*. The surface of *D. marginata* has developed an integrated system of multiscale channels so that the water spreads quickly and is transported via these channels very efficiently. These integrated multiscale channels have also been replicated using a facile soft lithography technique to prepare biomimetic surfaces and it has been proved that it is the surface architecture that plays a role in the water transport rather than the material’s properties (waxes present on the surface of the leaves). Based on our studies, we infer that the microlevel hierarchy of the structures make the surface hydrophilic and the multiscale channels allow the efficient passage and transport of water. The understanding of the efficient and well-directed water transport and collection in *D. marginata* is expected to provide valuable insights to design efficient surfaces for fog-harvesting applications.

## 1. Introduction

Water scarcity is a major problem in many parts of the world and often leads to health problems associated with drinking water from polluted sources [1]. Currently, billions of people still do not have access to clean drinking water. The experts say that the lack of clean water sources available will be the main issue in the coming years, especially in places such as Africa and cold desert regions in the Himalayas, where such problems could lead to mass migration of people to other areas of the world and subsequently may lead to an increase in social conflicts [1]. Many groups worldwide are working hard to find new sources of water which can satisfy the increasing needs of the population [2,3]. Rain, fog, mist, and dew are all sources of airborne moisture and all are important in the field of water-harvesting. Fog and dew both emanate from water vapor in the air condensing to form water droplets, either suspended in air, or formed onto a surface [4,5,6]. As conventional sources of water are depleting, the sensible and feasible method to harvest water is to collect fog and convert it into drinking water.

To survive in xeric conditions, nature has provided living species with methods of survival and there are many examples of flora and fauna absorbing airborne moisture which have been adapted according to their needs [7,8]. There are many studies directed in this field which shed light on how flora and fauna in arid regions are able to absorb airborne moisture and how their surface structure is responsible for the facilitation of the fog-harvesting process [8,9]. A significant amount of work has been carried out on plants with interesting surfaces, which can lead to a surface template for fog-harvesting. Mostly, these structures can be related to the native environment of the plants. For example, in the case of the Namib Desert grass *Stipagrostis sabulicola* the hierarchal arrangement of the surface microstructures present in its leaves aid in the collection of water from mist in lieu of water uptake from the soil [8]. Rosette plants have also been investigated for their ability to collect water from fog and low rain but they are not very efficient [10]. Cactus species such as *Opuntia microdasys* were explored for their fog-harvesting properties which rely on water transport through clusters of trichomes and conical spines arranged on the cactus stem [11]. In one of our previous works, we investigated the fog-harvesting mechanism of the well-known Bermuda grass *Cynodon dactylon* wherein we proposed that the combination of multilevel structures aids in the deposition, growth, retention, transport and detachment of water droplets [9]. One of the best examples of fog-harvesting in nature can be found in some beetle species in the African desert that use their body surfaces as fog collectors, a behavior referred to as fog-basking. These beetles adopt a head standing position facing the wind to collect fog droplets on their forewings, which are smooth with regular grooves that direct water droplets towards the mouth of the beetle. Droplets accumulate until they are large enough to coalesce and roll into the beetle’s mouth in which the orientation of the surface microstructures plays an important role in water collection [12]. Several lizard species (*Moloch horridus, Phrynocephalus arabicus* and *Phrynosoma cornutum*) found in arid and semi-arid areas have developed special abilities to collect water with their bodies’ surfaces and to efficiently transport the collected moisture for ingestion [13]. All three lizards have a honeycomb-like microornamentation on the outer surface of their scales and a complex capillary system in between the scales. Within this capillary system, the water is transported to the mouth where it is ingested. Spider silks have also been reported as excellent fog harvesting surfaces. The reported research in this field demonstrated the formation and collection of water droplets on the *Uloborus walckenaerius* spider’s web [14]. It has been demonstrated that silk webs are made from hydrophilic, moisture-sensitive proteins that enhance its wettability and form special microstructures in spider silk, which favors the condensation and collection of water drops.

Nowadays, scientists around the globe are trying to borrow the water collection strategies employed by different creatures in nature, using biomimetic principles [15,16,17,18]. The main goal of using these biomimetic techniques is to create such surfaces, which can be implemented in the design of fog-harvesting devices towards solving the growing global water shortage. The main issue to address is the efficient capture of water from either moisture or from airborne sources such as fog. Taking inspiration from the hydrophilic–hydrophobic patches of the *Stenocara* beetle, native to the Namib Desert, and the periodic spindle knots from spider silk; different fabrication methods have been developed to mimic these natural structures For the fabrication of hydrophobic–hydrophilic patches, replication techniques including lithography [19], patterning [20], printing techniques [21], etc. have been used; for the fabrication of fibers with a microlevel hierarchy, electrospinning is the main tool [22]. The understanding and replication of such surfaces can lead to everyday technological applications such as self-cleaning architectures based upon different natural surfaces having multilevel hierarchy which has been introduced into the paint, glass, automotive, and textile industries. Almost all reports that exist in the literature are focused towards the condensation and collection of fog on a surface. In addition to engineering the surfaces displaying superior water condensation on functional surfaces, it is also very important to address the issue of the transport and collection of captured water. The studies on the efficient transportation onto surfaces for fog collection still fall short.

In this work, we report on the fog-harvesting and water-channeling properties of a herbaceous fern, *Dryopteris marginata*, which inhabits moist humus-rich forest floors, under shade and is a native of the Himalayan regions of India, China, Bhutan, Nepal, Northern Burma, Tibet and Yunnan at altitudes less than 2400 m [23]. It cannot be grown throughout the world but can be exploited as a template for biomimetic fog-harvesting structures. In addition to investigating the fog-harvesting and water-channeling properties of this plant, we have also replicated the surface of its leaves on a polymeric material using soft lithography to prepare biomimetic surfaces and have demonstrated its utility for water capture and transport.

## 2. Experimental

### 2.1. Materials

For the fixation of fresh leaves, glutaraldehyde, potassium dihydrogen orthophosphate, di-sodium hydrogen orthophosphate and glycerol were purchased from Sigma Aldrich, Bangalore, India. For the replication of the leaves via soft lithography, polyvinylsiloxane (PVS), Coltene light body (ISO 4823, Type 3) was purchased from E-dental, Mumbai, India and epoxy resin was purchased from Araldite, Mumbai, India. The polyethylene sheet used for the control experiments as a flat surface was purchased from Sigma, Bangalore, India. All the chemicals were used as received. All the stock solutions were prepared in ultrapure water (18.2 MΩ cm) from double-stage water purifier (ELGA PURELAB Option-R7, High Wycombe, Buckinghamshire, UK). The general solvents for cleaning purposes such as ethanol and acetone were supplied by Merck, Bangalore, India. Sandpaper (150 grit) for the fixation of the natural leaf substrates was purchased from 3M, New Delhi, India. The *D. marginata* fern was collected from the Shimla region, India (31.1048° N, 77.1734° E) in the month of June (summer season).

### 2.2. Replication of Dryopteris marginata

The replication of surfaces of *D. marginata* was performed using a facile soft lithography technique optimized in our previous work [24]. In brief, the pinna of the fern was cleaned with a soft stream of deionized water to remove any dust particles. Then, the sample was mounted over a flat glass slide with the help of double-sided tape. Polyvinylsiloxane was applied on to the surface followed by the application of minor pressure onto the surface to obtain the negative replica. To obtain the positive replica, the epoxy resin and hardener were mixed in an optimized ratio and applied over the surface of the negative replica. The obtained substrates were dried in ambient conditions in a desiccator and preserved in a clean environment before the final use.

### 2.3. Scanning Electron Microscopy

The samples of the leaf for scanning electron microscopy (SEM) were prepared using the method described elesewhere [25]. The apex part of the samples was cut in such a way that all the main parts are available for analysis before fixing them for 2 h using the 2% glutaraldehyde in phosphate buffer (>99.0%). Then the samples were glycerol-substituted according to the procedure previously reported and dried in the desiccator for 24 h before SEM analysis. No gold coating on the samples was required before the analysis due to the conductive nature of glycerol. The samples were mounted on the carbon tape which was fixed to the circular aluminum stub. The surface analysis was done using a scanning electron microscope (FEI Nova Nano 460, Hillsboro, OR, USA) operating at 5 kV.

### 2.4. Water-Channeling Experiments

**Figure 1:**
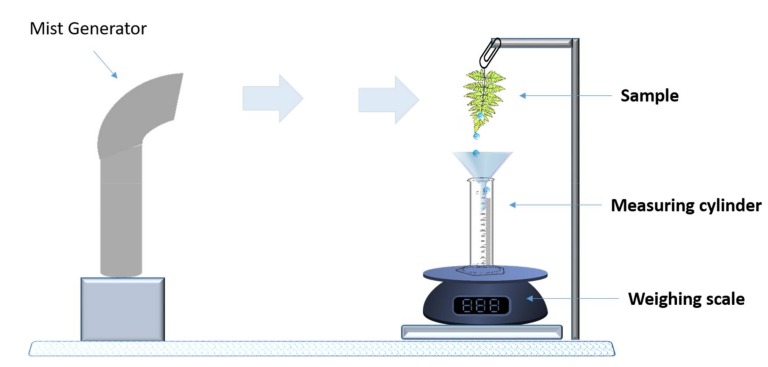
Schematic of the experimental setup for water collection.

Water collection experiments were performed by exposing the samples with a mist of ultrapure deionized water (18 mΩ) using a cold mist humidifier (Bionaire BU1300W-I, Lachine, QC, Canada) with an air flow of 130 ± 30 mL h^−1^ and at 15 cm from the sample. Weighing measurements were performed using a weighing scale (KERN, model: 440-43N, Balingen, Germany) with a 0.1 g resolution. The schematic of the experimental setup is shown in Figure 1. 

### 2.5. Statistical Analysis

All the experiments related to contact angle measurements and water collection studies were performed in triplicate. The obtained results were used for calculating the mean and standard deviation using the *n −* 1 method in Excel version 2015 software (Microsoft Office, Redmond, WA, USA)*.* The data was plotted using the OriginPro 8 software (OriginLab, Northampton, MA, USA)*.*

## 3. Results and Discussion

**Figure 2:**
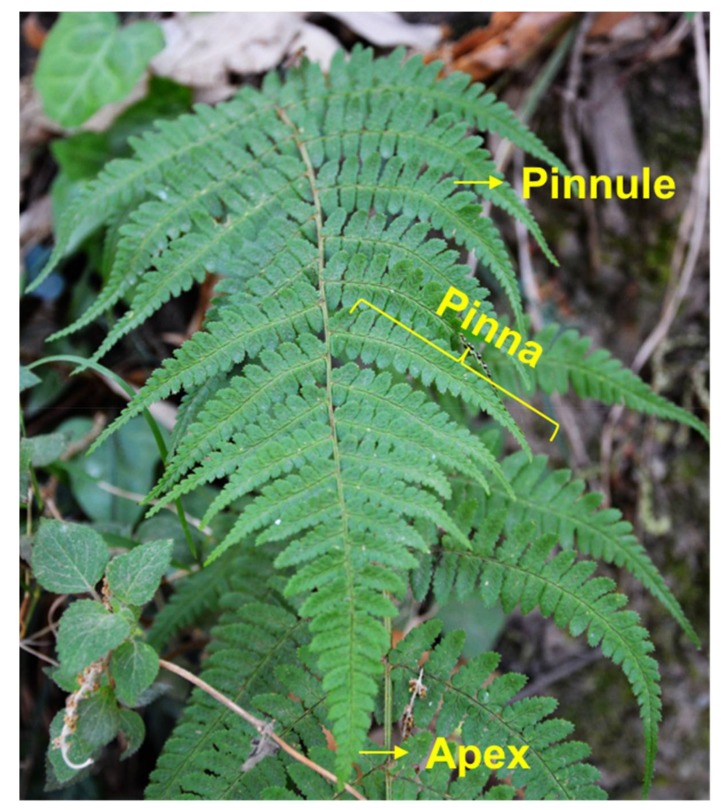
Photograph of *Dryopteris marginata* fern in its natural habitat.

*Dryopteris marginata* ferns are herbaceous having fronds approximately 20–25 inches long (Figure 2). The blade is 2–3-pinnate, pale green, broadly triangular lanceolate, widely truncated at the base and is about 65 × 25 cm. There are about 15–20 pairs of pinnae, alternate triangular lanceolate, approximately 25 × 6.5 cm in size and fibrillose on the costae. Pinnules are divided up into 15 lobes and alternate, shortly petiolate with an area of approximate 6 × 2.5 cm having an acute apex (Figure 2). There are small scales all over the leaf surfaces. The fern frond has a remarkable ability to channel water rapidly (downwards as well as sideways towards the apex of the pinna and the apex of the main blade which is gravity oriented).

**Figure 3:**
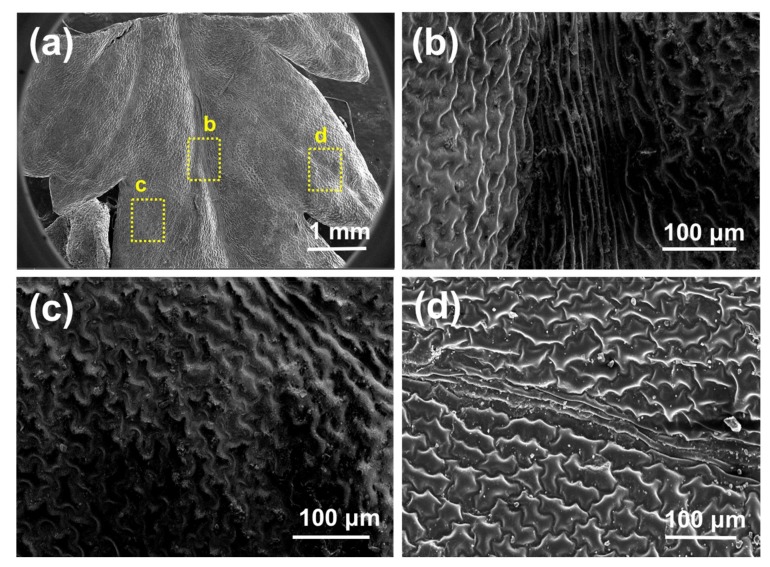
Scanning electron microscopy micrographs of a *D. marginata* leaf showing (**a**) the apex part of the pinnule, (**b**) the main channels, (**c**) the semicircular grooves and (**d**) the side channels.

**Figure 4:**
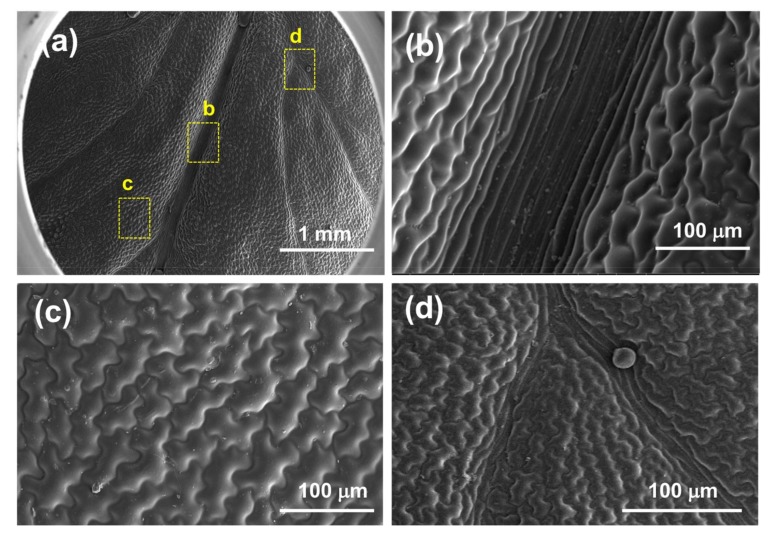
Scanning electron microscopy micrographs of a replicated *D. marginata* leaf showing (**a**) the apex part of the replicated pinnule, and (**b**–**d**) the replicated main channels, semicircular grooves and side channels, respectively.

Scanning electron microscope micrographs of the *D. marginata* leaf (Figure 3) show the complex hierarchical microstructure of the leaf surface. There are two types of structures present in the pinnules of the pinna revealed by SEM. Figure 3b shows the central part of the pinnule which runs to the tip having well-defined ridge-like channels. The channels are approximately 5–15 µm wide and lead to the apex of the pinnae where the water from the pinna coalesces. These deep trough-like channels are not very well defined but run parallel to each other channeling the water efficiently. These channels also connect to the inner axis and transport water to the central stipe which is gravity oriented. Figure 3c shows the side part of the pinnule apex which presents semicircular groove-like structures as compared to the different channel-like hierarchy shown in Figure 2b. These microstructures tend to extend from the edge of the pinnule to the central channels. These semicircular grooves, along with other microstructures, feed the water to the central channels, which either transport the water to the apex where it coalesces to form a droplet or to the central stipe which runs along the main blade to further converge the water to the main apex of the blade. Figure 3d shows the side part of the apex of the pinnule with narrow channels. These channels originate from the main central channels and run throughout the surface. To examine whether the water-channeling properties of the *D. marginata* leaf depend on the microstructure or material properties of the leaf surface, we replicated the surface of the fern frond using a soft lithography method on a polymeric material for the preparation of a biomimetic surface. Figure 4 shows the replicated surface of the *D. marginata* leaf and it is clearly evident that all the surface microstructures including the channels are efficiently replicated. From the SEM analysis, it can be concluded that there is a good degree of accuracy in the replication.

**Figure 5:**
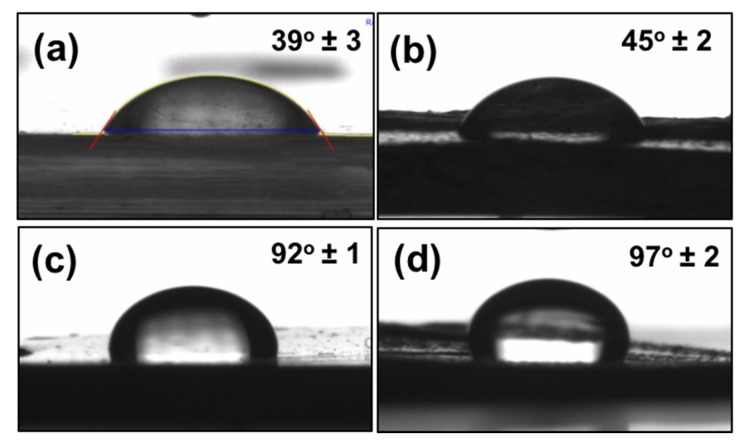
Contact angles of water (**a**) on the *D. marginata* leaf surface, (**b**) replica of the *D. marginata* leaf surface, (**c**) flat epoxy surface (non-structured) and (**d**) control polyethylene sheet.

To measure the wetting properties of the control surface, the leaf and its replica, we performed contact angle analysis (Figure 5). The analysis was performed using a commercially available contact angle analysis setup (Phoenix 300, Surface Electro Optics, Suwon-si, Gyeonggi-do, South Korea). The apparent contact angle measured on the *D. marginata* leaf was 39 ± 3°, on its replica was 45 ± 2°, and the contact angle measured on the control surface was 97 ± 2°. Taking measurements on the channeling samples was rather challenging as the water spread very rapidly on to the surface. It is noteworthy to mention that the contact angle was similar throughout the surface of the fern and its replica. Under fog conditions, water droplets efficiently channel along the length of the leaf towards the ground. The water droplets channel preferentially (asymmetric anisotropic) along one direction towards the apex. The surfaces display hydrophilicity, with the fern and its replica exhibiting low static contact angles (39 ± 2° and 45 ± 2°, respectively) and high hysteresis values (15° and 21°, respectively) (Table 1). It was interesting to observe that while the epoxy replica of the silicon wafer and the control polyethylene sheet had static contact angles well above 90° (92 ± 1° and 97 ± 2°, respectively), which are significantly higher than the real *D. marginata* leaf (39 ± 2°) and its replica (45 ± 2°), the control surfaces also showed low hysteresis values (5° for the flat epoxy surface and 6° for the polyethylene surface) as compared to the *D. marginata* leaves and its replica. From this observation, it can be concluded that the surface microstructures (i.e., semicircular grooves and microchannels) are responsible for the wetting properties of the leaf surface and its replica.

**Figure 6:**
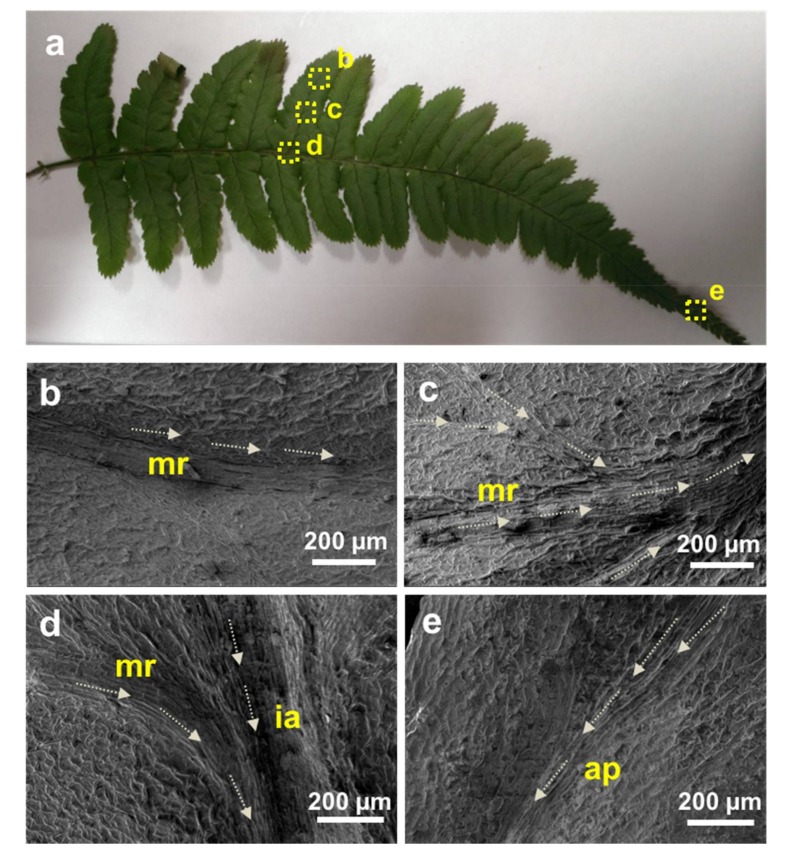
**(a)** Photograph of the *D. marginata* frond with labeled areas showing different locations of the water channels. (**b**–**e**) Scanning electron microscopy micrographs of the labeled areas in (**a**); arrows indicate the presumed direction of the water transport through these channels. ap: apex; ia: inner axis; mr: mid-rib.

Figure 6a shows the location of the different types of channels present on the fern frond. The SEM images of the different types of channels are shown in Figure 6b–e. Figure 6b shows the channels located at the center of the pinnule, which runs along the axis to the costa (mid-rib). Figure 6c shows the small channels which look like the tributaries distributed at the different locations on the pinna forming interconnected networks that merge to the channel shown in Figure 6b. Figure 6d shows the channels located at the costa (mid-rib) where the channels from the pinnule merge. The water moves from the pinnule channels to the costa channels, which ultimately merge to the central inner axis (rachis) channel. The water flows along these channels and moves under gravity, and ultimately coalesces at the tip of the apex through the channels shown in Figure 6e.

**Figure 7:**
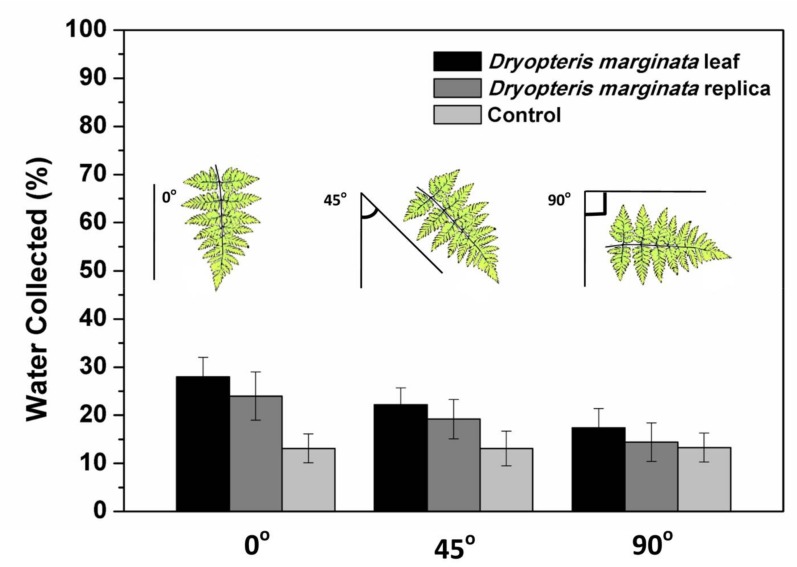
Water collection efficiency of the *D. marginata* leaf surface, its replica, and the control sample at different angles. Data is shown as the mean ± standard deviation.

The fog-harvesting and water-channeling experiments were conducted on different samples to show the exceptional water collection behavior of the *D. marginata* leaf (see Figure 1). A polyethylene sheet, devoid of any surface features, was used as a reference sample for control experiments. Figure 7 shows the results obtained from the fern frond, its replica, and the control sample. The area of the control sample was normalized by calculating the leaf area using a suitable imaging software, such as ImageJ software [26]. The leaf, the replica and the control samples were exposed to cold water mist at angles 0°, 45° and 90°. The water was collected according to the setup shown in Figure 1 and the efficiency of the channeling was calculated by the amount of cold mist exposed to the samples and the final volume of the water collected. We calculated that the maximum efficiency obtained was approximately 28% when the fern samples were exposed at an angle of 0°. The efficiency decreased when we changed the angles towards the horizontal position and the efficiency was 22% and 17% for the 45° and 90° orientations, respectively. This can be possibly attributed to the orientation of the microchannels towards the apex as demonstrated by SEM analysis.

**Figure 8:**
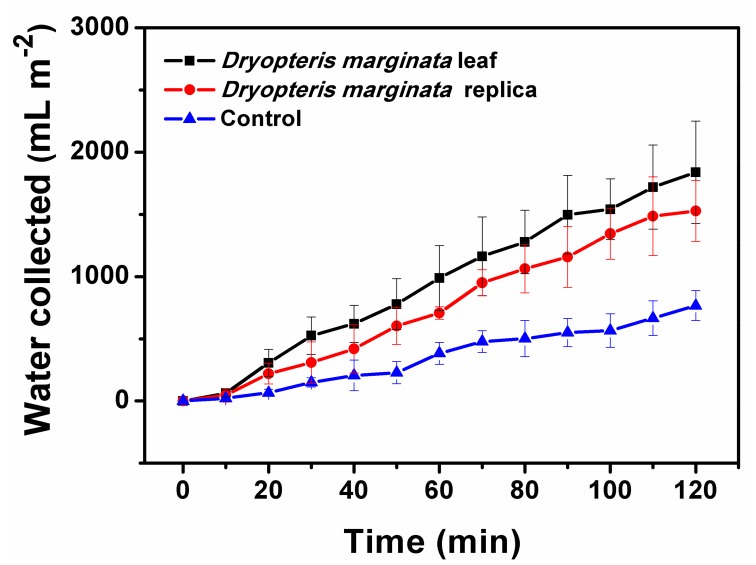
Water collection dynamics of the different substrates. Amount of water collected by surfaces exposed to fog flow at intervals from 0 to 120 min with 10 min increments, shown as the mean ± standard deviation.

Water-channeling was observed as soon as the surface of the leaf and its replica were wetted by cold mist. To study the water-channeling dynamics, the samples were subjected to the mist for two hours and the amount of water collected was measured. During two hours under mist conditions, the leaf collected 68 ± 8 mL of water. Similarly, the water collected from the leaf replica was 52 ± 9 mL. The water run-off dynamics of both the real and replicated substrates were almost consistent and water collection was almost consistent and at a steady-state with respect to time (Figure 8). The overall amount of water channeled on the real leaf and the replica of *D. marginata* occurred at a steady rate that fit a linear regression model (*R*^2^ = 0.96 and *R*^2^ = 0.89, respectively). Compared to previously described fog-harvesting systems, substrates based on the *D. marginata* leaf showed very good water collection efficiency per unit area and the water was collected at a steady rate, which makes them very good candidates to be utilized in fog-harvesting devices [8,11,16,22,27,28].

Generally, the fog collection process consists of three stages: the fog transport onto the surface, the capture of fog droplets on the surface, and the removal of the captured water from the surface. In the process of fog collection, many tiny water droplets in air are convected towards the surface, directly collide and are captured on the surface. Due to a small inertia of the colliding droplet as well as adhesion between the incoming droplet and the surface, most of the incoming droplets will be captured onto the surface irrespectively of the surface wettability. In addition, the moisture present in the mist will be captured via condensation on the surface, which depends on the surface wettability. Therefore, in this process, the water removal process is the most important step in determining the efficiency of a fog-harvesting substrate.

**Figure 9:**
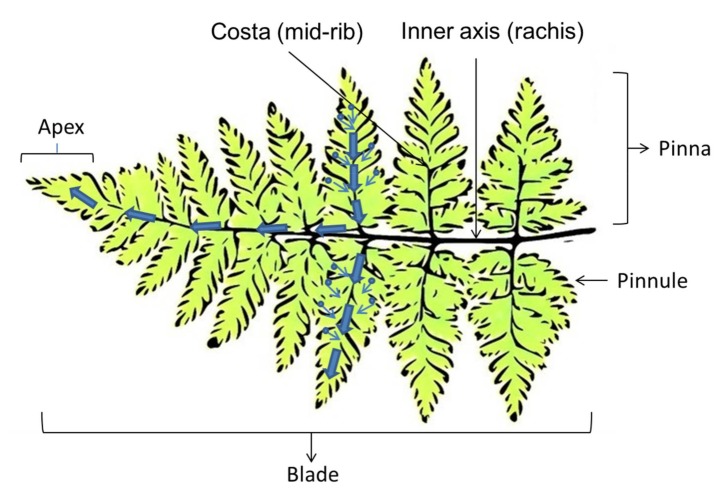
Schematic showing various parts of the fern leaf and the presumed directions of the water transport, which is based on the orientation of the leaf and gravity.

Figure 9 shows the schematic of the fern frond depicting the possible directions of the water transport. From the data presented here, it is clear that the channels are responsible for the efficient water removal from the surfaces. Generally, the water flow in a capillary system is described by the Washburn equation which depends on the geometry and width of the microchannels [29]. In this case, the movement of the water through the microchannels in both the real and replicated *D. marginata* leafs can be predicted by capillary and gravitational forces. From the experimental data (as presented in Figure 7), it is clear that the gravitational force has a significant contribution in addition to the capillary force. As the surface of the leaf and the replica is hydrophilic, water is adsorbed efficiently on to the surface and due to the forces mentioned above, is channeled very efficiently towards the apex where the channels end.

The transport mechanism is almost homogeneous. When the capillary system is initially filled, the water movement through the capillary is even swifter. This can be confirmed from Figure 8 where the initial water collection is low, shows a cascading behavior and follows a steady flow afterwards. In the control samples this type of flow cannot be seen, which confirms that the surface morphological features of the *D. marginata* leaf, including the network of microscopic channels and the semicircular grooves, are responsible for the directed flow and the efficient channeling of water. It is noteworthy to mention that the surfaces based on *D. marginata* are very efficient for water collection and on a par with other reported bioinspired surfaces as illustrated in Table 2. In summary, the surfaces inspired by *D. marginata* offer very good fog collection efficiency per unit area at a consistent rate. The knowledge and the findings based on these efficient water-collecting surfaces can pave way for the design of highly efficient surfaces to be utilized in fog-harvesting and other industrial applications.

## 4. Conclusions

In pursuit of the design of efficient channeling surfaces for application in fog-harvesting devices, the fog-harvesting and -channeling properties of the *D. marginata* leaf were investigated. The surfaces of *D. marginata* have a remarkable ability to channel water rapidly and efficiently, which can be attributed to the integrated system of the multiscale channels and surface microstructures. The surface of the real leaf was replicated using a facile soft lithography technique and good channeling properties in the replicated surfaces proved that efficient water-channeling is due to the surface microstructures rather than the surface chemical composition. The experimental data suggested that collected water is efficiently transported by an interscalar capillary network and the mechanism of this transport has been discussed. This understanding of efficient and well-directed water transport and collection due to the interscalar network of the microchannels in the *D. marginata* leaf provide a promising approach to design efficient surfaces for application in fog-harvesting.

## Figures and Tables

**Table 1:** Water contact angle mean values for the adaxial surface of the *D. marginata* leaf, its replica and control surfaces.

Substrate	Static Contact Angle	Receding Contact Angle	Advancing Contact Angle	Contact Angle Hysteresis
*D. marginata* leaf surface	39°	29°	44°	15°
Replica of *D. marginata*	45°	28°	49°	21°
Flat epoxy surface (non-structured)	92°	90°	95°	5°
Control polyethylene sheet	97°	95°	101°	6°

**Table 2:** Comparison of fog-collection performance of different bioinspired surfaces reported in the literature.

Bioinspired Surface	Material Used	Fog Collection (g cm^−2^ h^−1^)	Ref.
Micropatterned superhydrophobic surface fabricated using inkjet printing	Polystyrene	≈0.06	[21]
Micropatterned bioinspired surfaces fabricated using photolithography	Polyurethane	≈1.69	[19]
Microstructured surfaces and mesh	Epoxy and Polyolefin	≈0.18	[16]
Biomimetic coatings on surfaces	Polystyrene	≈3.40	[30]
Bioinspired hybrid micro-/nanopatterned surface	Polytetrafluoroethylene	≈0.2	[28]
Bioinspired surfaces with star-shaped wettability patterns	Heptadecafluorodecyl-trimethoxysilane/TiO_2_	≈2.78	[31]
Heterogeneous rough conical wires	Cu wires	≈0.6	[27]
Bioinspired micropatterned surfaces fabricated using photolithography	Hexamethyldisiloxane/Si	≈1.12	[6]
Natural hierarchical surfaces	Natural wax/cellulose	≈0.96	[9]
Modified Raschel mesh	Steel mesh with superhydrophobic coating	≈3.40	[32]
*D. marginata*-based patterned surfaces	Natural leaf/epoxy polymer	≈0.72–1.1	This work
